# Adverse Human Health Impacts in the Anthropocene

**DOI:** 10.1177/1178630218812791

**Published:** 2018-11-16

**Authors:** Richard Olawoyin

**Affiliations:** Department of Industrial and Systems Engineering, School of Engineering and Computer Science, Oakland University, Rochester, MI, USA

**Keywords:** Anthropocene, child, particulate matter, air pollution, heavy metals, Wilms tumor

## Abstract

This commentary presents a summarized discussion of key findings and relevant ideas from previously published study, index analysis, and human health risk model application for evaluating ambient air-heavy metal contamination in Chemical Valley Sarnia (CVS). The CVS study provides previously unavailable data in the CVS area which evaluates the adverse effects on air quality due to nearby anthropogenic activities. The study provided an assessment of environmental pollutants, finding that carcinogenic and non-carcinogenic substances are present in trace quantities. The main findings of the study suggest that chronic exposure of humans to several contaminants identified in the area studied may lead to carcinogenic health effects, including cancer (such as nephroblastomatosis) as well as non-carcinogenic health effects, such as damage to the tracheobronchial tree. Children were found to have a significantly higher risk, that is, a higher hazard index: a value used to measure non-carcinogenic health risk from heavy metals identified in air samples collected during the research period from 2014 to 2017. This study quantified the influence of environmental contaminants, relative to human exposures and the consequence of developing nephroblastomatosis in the human population.

**Comment on**: Olawoyin R, Schweitzer L, Zhang K, Okareh O, Slates K. Index analysis and human health risk model application for evaluating ambient air-heavy metal contamination in Chemical Valley Sarnia. *Ecotoxicol Environ Saf*. 2018;148:72-81. doi:10.1016/j.ecoenv.2017.09.069.

## Introduction

The advent of technological advancement and innovation has yielded enormous benefits supporting industrialized economies and the development of the new industry known as “Industry 5.0”—the industry of the future with focus on personalization and customization of products and services for all. Industry 5.0 also increases the collaboration between machines and humans working side by side for optimal productivity.

The expansion in human activities has been the dominant influence on the environment, including the human environment, in a period known as the Anthropocene. Environmental pollutions affecting the ecosystems and human health indicate an increase in stress response among the individuals impacted. In an extant study,^1^ the vulnerability of children and families, and communities, to the psychological, social, economic, and ecological consequences of environmental degradation from anthropogenic activities was found to extend beyond the initial periods of the disasters. The strongest predictors of stress were family health concerns, commercial ties to renewable resources, and concern about economic future, economic loss, and exposure to the contaminants. Upsurge in the levels of contaminants generated and released into the environment from anthropogenic activities such as industrial or manufacturing processes is problematic and will continue to arouse public attention.

Environmental pollution in the Anthropocene has adverse toxic effects on human health depending on the concentration and dose of the chemicals of exposure relative to the location of the receptor. A conventional deterministic risk assessment method can be used to estimate the potential carcinogenic and non-carcinogenic risks from worst-case inhalation, ingestion, and dermal contact exposures to chemicals of potential concern (CoPC), in the air, food, water, and soil for susceptible population in polluted areas such as the Chemical Valley Sarnia (CVS).

**Figure 1.**
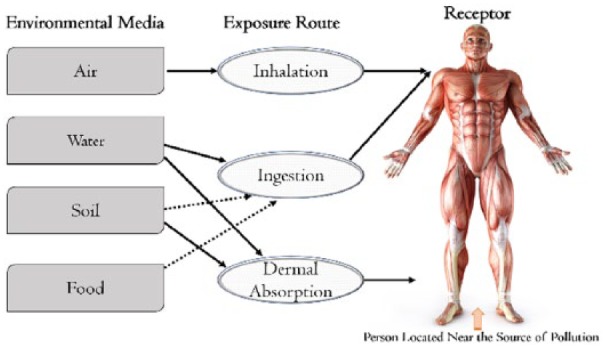
Human exposure to potential CoPC through multi-pathways. CoPC, chemicals of potential concern.

The route of entry of toxic pollutants into the human body can be through 3 main exposure pathways: (1) inhalation of particles present in the air, (2) ingestion, and (3) dermal absorption as shown in Figure 1. The ingestion exposure could occur through eating of soil particles and/or licking contact surfaces (eg, hands). Dermal absorption could occur through exposed skin, whereas particles are inhaled through the mouth and nose during breathing activities.

Inhalation is a major exposure pathway for volatile organic compounds (VOCs) and particulate matters with impregnated toxic heavy metals. The presence of such pollutions near human habitation agitates the public due to the potentially hazardous nature of the pollutants. Both acute and chronic exposures to CoPC are critically important for the assessment of the potential impacts to human health. Unfortunately, public attention is often not garnered and maintained because of the intricacy of incremental degradation of human health from long-term chronic exposures. This subtle and potential source of exposures to familiar substances such as trace amounts of metals may pose significant public health hazards but too often remain largely unidentified.^2^ Environmental research is effective in providing appropriate identification, assessment, and characterization of pollutants, which will in turn help protect the public from environmental pollution hazards, especially in the Anthropocene.

The CVS impact assessment provides benefits to the community by presenting the findings of the study to the public and provided significant conclusions that can help promote human health protection for the residents of the area. The study considered the following:

1. Environmental pollution effects on human health from air quality degradation in the region;
2. Assessment of human exposure (chronic and acute) to hazardous contaminants based on comprehensive assessment of exposure for both adults and children;
3. Risk level assessment and characterization of the prevalence of nephroblastomatosis (Wilms kidney cancer) in the area;
4. Risk assessment and determination of other carcinogenic and non-carcinogenic health effects based on the impacts of human exposure to the pollutants based on the quantities identified during the study.

## Fate and Transport of Pollutants in the CVS

Considering the significance of the area of research selected for the CVS study, it is also important to evaluate the heavy industrial activities on the waterfront of the Sarnia. Located at the mouth of Lake Huron, which provides significant coastlines of Michigan and the Ontario Province, Sarnia Canada also sits along the St Clair River which borders between Ontario and Michigan and flows into the nearby Lake St Clair.^3^

The river continues its flow out as the source of the Detroit River, feeding directly into Lake Erie which provides coastlines for Ohio, Pennsylvania, and New York. The CVS study provides Sarnia with important data to better understand anthropogenic sources of atmospheric pollutants generating hazardous air quality conditions which are certain to be a source of atmospheric deposition of these contaminants to surrounding populations.

**Figure 2.**
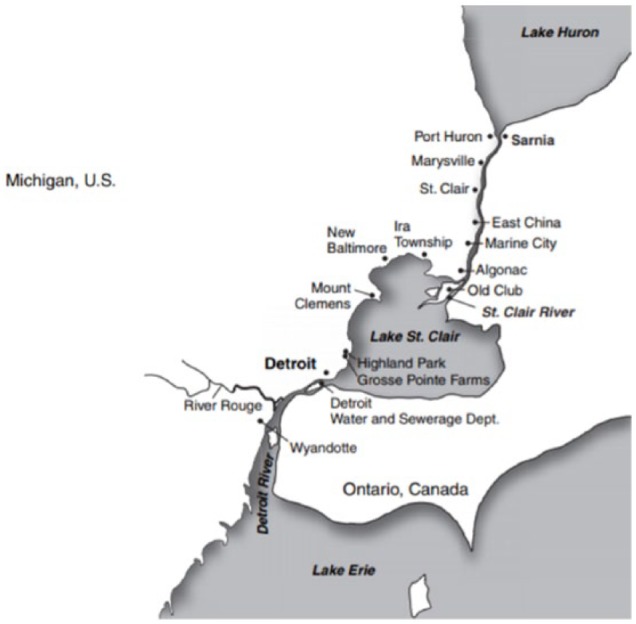
Map of the study area.^3^

**Figure 3.**
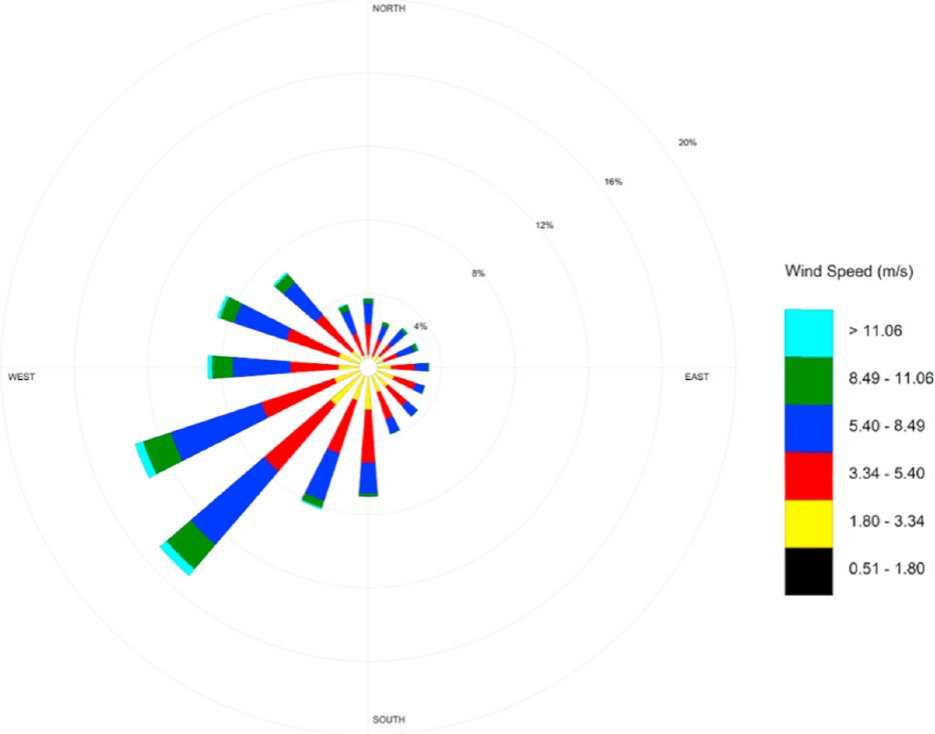
Wind rose diagram of the CVS area.^1^ CVS, Chemical Valley Sarnia.

**Figure 4.**
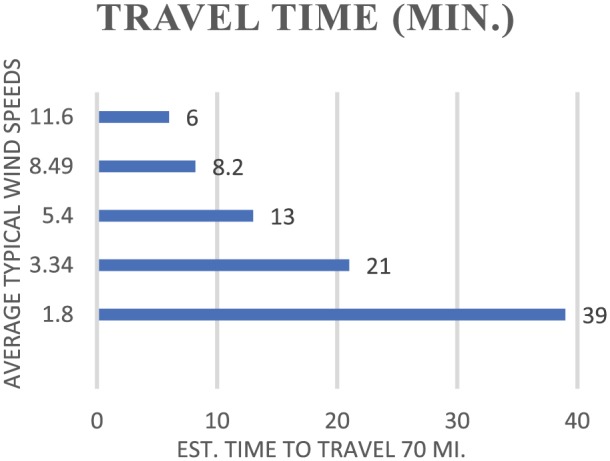
Estimated travel time of airborne pollution.

Sarnia is located at the top center position (upwind), with potential of generated pollutants reaching sufficiently high altitudes and dispersed downwind toward the Detroit Metropolitan Area (see Figure 2). When compared with Figures 3 and 4, airborne pollutants from Sarnia are likely to make the less than 70-mile journey very rapidly as pollutants are carried by the prevailing southwesterly winds traveling at the approximate speed of between 3.5 and 10 miles/h.

The surrounding environment and populations sharing local waterways and proximity with Sarnia are where contaminants mostly accumulate with potentially adverse effects within aquatic biomes, including the ocean as the water of the Great Lakes terminates in the Atlantic. Direct contamination of waterways from industrial activities in Sarnia is a recurrent event. An example is the 2004 river contamination, when an estimated 39 000 gallons of methyl ethyl ketone and methyl isobutyl ketone were discharged into the St Clair River.^4^ This single chemical spill affected more than a dozen water plants, shown in Figure 4, within Canada and the United States from which about 36 000 customers access and consume water.^4^

The CVS study is quintessential due to the proximity of the heavy industrial activities along Sarnia to multiple densely populated urban locations. Potential exposures to hazardous air conditions of suburban areas were considered in the study and the diminished air quality around these locations. Urban centers continually generate their own ongoing source of airborne contaminants including lead from sources such as leaded fuel and leaded paints or incinerated refuse.^5^ In effect, anthropogenic pollutants released into the atmosphere in Sarnia only need to be transported short distances before they are compounded as they become available for deposit in Cleveland, Toledo, or at other surrounding suburbs.

The travel time by which environmental airborne pollutants can be transferred across locations such as from Sarnia to Detroit is illustrated in Figure 4. The range of typical southwesterly wind speeds is divided into a conservative distance of travel between the 2 locations.

A comparison of the wind and travel time is shown in Figure 4. Even during periods of slowest wind speeds, airborne pollutants can reach densely populated areas of downwind in fewer than 2 days. Although more typically this distance will take far less than half this amount of time, less than 18 hours on average. Clearly, among the most important ideas related to the CVS study is the chosen location.

## The Importance of Air Quality at CVS

An air quality study quantifying the concentration of anthropogenic pollutants in the region had not been provided until the CVS study.^3^ Thus, it is a noteworthy idea as a location of research, given the potential benefit to improve human health of the at-risk population in the CVS area. The research study provides crucial information from which to determine the sufficiency of air quality according to standards for harmful pollutants set by the US National Ambient Air Quality Standards (NAAQS) of the United States Environmental Protection Agency (US EPA).^3^ Based on the level of air quality as assessed during the air quality analysis study in the CVS area, the populations represented within the studied area and their municipalities can, for the first time, accurately begin to understand the human health risks posed by anthropogenic environmental contaminants, primarily from activities of the significant presence of the petrochemical industries located in Sarnia.

Time-weighted average dose was linked to the exposure simulation concentrations for heavy metals and this was used for the exposure analysis for the inhalable particulates. The assessment of the chronic and acute inhalation exposure risks considered the dose-response criteria of the US EPA and the carcinogenic and non-carcinogenic risks for all risk-posing CoPC were estimated for the different population groups. These activities included collecting and analyzing air samples from multiple locations during a 3-year span of the CVS study, and the resulting data that were analyzed offer further benefits as a guide to organizing efforts tasked with mitigating hazardous exposure to environmental contaminants. By providing data on the distribution of contaminants at sampled locations, the CVS study contributes to the body of knowledge necessary for identifying and mitigating the pollution debacle in the region. Cleanup efforts which strategize hazard mitigation according to the available data maximize the health improvements which can be made on behalf of the at-risk population. In addition, the CVS study can be used as a tool for understanding the fate and transport of hazardous materials in the region and evaluate the rate of human exposures to the pollutants of concern.

## Applied CVS Study Methods

To achieve the study objectives, an ecological risk index assessment was conducted on the collected air samples to estimate the deposition fractions and deposition fluxes in the human respiratory system. The study assessed the effects of the trace-metal-bound particles and the penetration potentials through the head airways and possible damages to the tracheobronchial tree and the alveolar region, thus leading to the development of nephroblastomatosis, especially among children.

The CVS study also calculated the contamination factors, enrichment factors, pollution load index, and contamination indices. Hazard quotient (HQ) is the ratio of exposure to the estimated daily exposure level at which no adverse health effects are likely to occur. Non-carcinogenic risk assessment of the CoPC was conducted and the HQs of the individual exposure from the heavy metals were determined. If HQ > 1, the receptor is at risk of non-carcinogenic effects. When HQ > 1, the risk of non-carcinogenic can result in adverse health damages to the human body. The HQs were calculated for non-carcinogenic parameters for inhalation pathways.

Incremental lifetime carcinogenic risks (ILCRs) from the heavy metals were also investigated. If the ILCR of the CoPC is more than 1×10^−4^, it is considered as “definite risk,” and the other risk levels are described in Table 1.

**Table 1.** The risk range of ILCR.

ILCRs	Human health risk level
<1×10^−6^	Acceptable/negligible
(1×10^−6^)−(1×10^−4)^	Potential human health risk
˃1×10^−3^	Serious human health risk

ILCR, incremental lifetime carcinogenic risk.

The study determined that the main route of entry of contaminants into the human body is through inhalation of respirable fractions of the contaminated-particulate-matter-bound heavy metals. These inhalable fractions of airborne particles are deposited in the respiratory tract along the airways during a single regular breath.^5^ To establish an ecological risk index, the CVS study provided the following assessments:

1. Assessment of contamination factors to determine the extent of environmental pollution from industrial activities and the contamination of air quality in the CVS;
2. Assessment of the pollution load index to estimate the contamination of the ambient environment for the use of comparing the assessed pollution levels between sample locations;
3. Assessment of the enrichment factor to determine if the concentration of the enriched sample is from natural deposits by geogenic (geological processes) or anthropogenic (man-made) sources;
4. Assessment of human health risk to determine exposure via inhalation of atmospheric particulate matter bound with heavy metals;
5. Assessment of the ILCR to determine the incremental probability of an exposed person to carcinogenic substances developing cancer in his or her lifetime;
6. Assessment of non-carcinogenic health risks provided with use of the target HQ and hazard index used to measure health risks of heavy metals found in airborne particles.^3^

The negative human health effects that may result from chronic exposures to the toxic metals selected for chemical and statistical analyses do include health risks which are important to consider. Through the application of the briefly described statistical methods, the CVS study concludes that anthropogenic heavy metals within sample particulate matter may be a potential source of the adverse effects leading to the concentration of the nephroblastoma disease in the area.^3^

## Credible Risks of Nephroblastomatosis

Between 1990 and 2009, 63% of occurrences of nephroblastoma occurred among children under 5 years of age.^3^ From 2000 to 2009, approximately 72% of these nephroblastoma cases occurred in Marine City, MI. This community located within 20 miles down the St Clair River from Sarnia, which was defined as a cancer cluster because of the high prevalence of cancer over time.^6^ The CVS study provides a characterization of nephroblastoma as a solid tumor of epithelial tissue formed from immature kidney cells which grows quickly on the exterior of the human kidney resulting in nephroblastomatosis.^3^

Nephroblastoma or Wilms tumor is the most common renal tumor in children with peak instances occurring at the age of 3 to 4 years.^7^ The prognosis of nephroblastoma is highly variable depending on its stage of development and has 2 histopathologic types, favorable and unfavorable, each having outcomes across the spectrum for each of these 2 categories.^7^ The cancer tumors are usually found in the adrenal gland, resulting in abdominal pain and distention. The tumors are mostly large in size. Ultrasound imaging procedures are an effective means of assessing internal tumors without subjecting children to harmful sources of radiation allowing medical staff to rule out occurrences of capsular or vascular invasion occurring in 4% to 10% of all patients.^8^

Treatment of the condition varies depending on the size of the tumor developed at the time of treatment as well as other considerations such as the bilateral presence of tumors on both kidneys.^9^ Other determinates for course of treatment include factors such as age, overall health, medical history, the extent of the disease, and tolerance to specific therapies. Common treatment also includes the surgical procedure of a radical nephrectomy which removes the kidney in its entirety, the surrounding tissues of the kidney, the ureter which drains urine from the kidney to the bladder, and the kidneys’ corresponding adrenal gland which is located on the top of the kidney.^9^ When the tumor presents on both kidneys, surgical treatment consisting of a partial nephrectomy is recommended. This surgery is intended to remove the entirety of the tumor, leaving behind the maximum amount of kidney tissue unaltered by the tumor in an effort to spare as much of the kidney as possible. Approximately 5% to 8% of children diagnosed with capital nephroblastoma developed tumors in both kidneys.^3,10^ In cases where treatments are difficult or impossible, to remove tumors affecting both kidneys, which are too large to perform a partial nephrectomy, patients will be subjected to radiation therapy in an attempt to shrink the tumors prior to surgical removal.^8^

In children, the prognosis for long-term viability of nephroblastoma survivors depends on many factors including the stage of the disease as well as the characteristics of the tumor cells, which may be assessed under the magnification microscope, the promptness of nephroblastoma identification and treatment, and the continued follow-up of medical care provided to the patient including ongoing adjustments to the course of treatment and continued nephroblastoma screenings.^9^ Over time, the overall success rate of medical treatment and the patient experience of treatment side effects have both improved significantly. For the past 20 years, the 5-year survival rate among children diagnosed and living with nephroblastoma is approximately 86% and the overall longter survival rate is even higher (up to 90%).^10,11^

Whereas the treatability and long-term survivability of the cancer may be a source of hope to patients and their health care providers, the dramatic courses of treatment required for nephroblastoma survivors merit a closer look into causative forces. The CVS study offers the populations affected and at risk of adverse health effects a clearer understanding of a potential source of pollutants resulting in the cancers prevalent around their communities. Among the results from the CVS study were the confirmation of the findings: (1) concentrations of airborne lead are present in air samples in excess of 350% above NAAQS air quality standards; (2) concentrations of chromium (VI), lead, and nickel are partly the result of anthropogenic activity; (3) ambient air is polluted in the study area with particle material with bound metals which may increase the human health risk of nephroblastoma, especially in children; (4) high risk of potential cancer affects children and adults in the area studied; (5) children are more likely to develop carcinogenic and non-carcinogenic health effects from exposures to elemental concentrations of airborne particulate matter.^3^

## Final Thought

The public health benefits of the CVS study are quintessential to the proper identification of anthropogenic pollutant sources, the concentration in the environment, and the potential effects on receptors exposed to the contaminants. Ideally, the prevalence of childhood nephroblastomatosis combined with the results of this study will help decision-makers provide adequate care for the residents of the CVS area and other adjoining areas.
